# The study of the separation and deposition of dredging soil slurry under physical disturbance

**DOI:** 10.1371/journal.pone.0281708

**Published:** 2023-03-09

**Authors:** Yan-zhao Yuan

**Affiliations:** Lecturer College of Civil and Traffic Engineering, Henan University of Urban Construction, Ping dingshan, China; Tribhuvan University, NEPAL

## Abstract

Most existing research uses experimental designs for testing, which cannot efficiently analyse the migration and sorting rules of particles in the disturbed slurry. Therefore, based on the fluidized bed flow film theory, a slurry flow film structure system is established according to the disturbance state of the fluid. On this basis, the particle size and distribution law of the disturbing force formed by slurry disturbance are analyzed, and the calculation model of single particle lift in the flowing film is also analyzed. On this basis, using Markov probability model, the probability of particle lifting and sorting between layers is theoretically deduced. Then, according to the particle ratio of the original mud, the settlement gradation of the particles in the disturbance is analyzed. It can also predict the separation degree of particle in natural turbulence, fluidized beds, and sludge mechanical dewatering. Finally, according to the particle flow software PFC (Particle Flow Code), the main influencing parameters (disturbing force and gradation) were verified and analyzed. The results show that the particle flow simulation results are in good agreement with the calculation results. The model of slurry membrane separation proposed in this paper can provide a basis for studying the mechanism of slurry disturbance separation and particle deposition.

## 1. Introduction

Large deposits of silt, including fine grains of sand and a large proportion of clay particles, are found in natural river basins and estuaries. Thus, a muddy soil layer is formed after deposition, which has the characteristics of poor permeability, weak strength, long consolidation period, etc. Therefore, vacuum preloading and soft soil solidification are commonly used when this kind of soil material is encountered in waterway silt discharge engineering and reclamation engineering. Nevertheless, the effect is poor, and the energy consumption is significant. Some Scholar. [1–3] studied the liquefaction process of seabed silty soil under the action of waves. After the disturbance of silty sand, the pore pressure in the soil layer oscillated, the resistance of the layer improved, and many clay particles were removed. The structure of the layer and the physical properties of the liquefied silt have been studied. The median particle size of liquefied mud increased obviously. At the same time, the clay content and water content decreased obviously. The strength and density of soil are effectively improved. [4], Some scholars. [5–7] proposed the idea of artificially disturbing liquefied silty soil. The test shows that the permeability, force and soil structure of the silt are effectively enhanced after artificial disturbance of the silt deposit. However, the migration law and physical property changes of mud caused by artificial irritation are primarily analysed from an experimental point of view, but few theoretical studies have been conducted. Only some scholars [8–11] have made in-depth discussions on the particle migration law of waves to riverbed from different angles, such as riverbed wave height, riverbed resistance and riverbed morphology, and further understood the movement law of sand waves. However, it is still lacking to combine probability statistics theory with sand wave motion research method to quantitatively study the migration law of disturbed particles, such as the flow field structure (particle motion state and migration process) and the probability of particle hovering position (internal seepage hovering or free fall hovering) after physical disturbance of silty mud.

In this paper, the slurry is regarded as a thick particle fluid. The separation film theory of viscous particle flow under the disturbance force is established by combining the theory of sediment start-up and migration with the theory of fluidized bed separation film. This theory effectively introduces the traditional mud law under the action of disturbance into the concept of forming a similar physical field. The disturbed slurry includes a flowing film with a density as the main factor, and the movement of particle settling expands from single-particle movement to group particle movement. The particles migrate from disordered turbulence to layered laminar flow for sorting. Combined with earlier studies, the physical concepts of density difference shift. The increased spin of the particles and the transfer of the pressure difference were achieved, further improving the accuracy of the formula of the particle model. A statistical Markov chain model suitable for particle migration between flow films was introduced, and the calculation method of particle size distribution and separation under disturbing force was theoretically derived. This method not only analysed the movement and the law of particulate suspension from the point of view of a single particle. However, it could also reasonably describe the filmogenesis of manure during the disturbance process, the migration of particles under the structural system and the relationship of density sorting.

## 2. Mathematical model

### 2.1 Mechanism of single-particle suspension of slurry under disturbance

**Fig 1:**
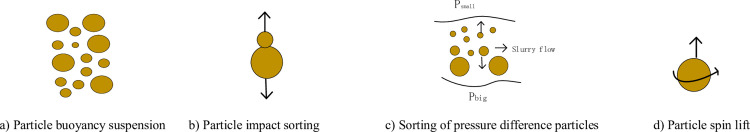
Motion state of flow film particle separation. a) Particle buoyancy suspension, b) Particle impact sorting, c) Sorting of pressure difference particles, d) Particle spin lift.

According to the study of the mechanism of the particles suspended in slurry during the disturbance, it is found that the stress state of a single-particle migration and separation consists of the following four states (as shown in Fig 1):

1. When the turbulent velocity in the vertical direction is greater than the gravity deposition velocity of the particles, the particles are in a suspended state, as shown in Fig 1A) [8].
2. Particle displacement caused by collisions and other factors caused by particles flowing with the slurry is shown in Fig 1B) [9].
3. In the process of flow, due to the action of the disturbing force, there is a pressure difference between the flow rate of the particle flow film and the flow rate of the external slurry, which leads to the suspension of the particle pressure gradient, as shown in Fig 1C) [9].
4. According to the Magnus effect, when particles are subjected to fluid pressure and shear stress, the particles themselves will produce relative spin motion with water flow and other environments. This movement will cause the rotational lift in the direction of particles, causing the particles to suspend and rise, as shown in Fig 1D) [10].

The suspension of fine particles is mainly caused by the collision between turbulent buoyancy and particles. In contrast, the floating of coarse particles needs to generate gradient pressure and spin lift to cause the suspension and separation of coarse particles [11].

The analysis of the particle sorting process under turbulent conditions shows that fine particles easily float up, and the suspension conditions of coarse particles are more complicated. Therefore, in the disturbance process, fine particles with lower density tend to concentrate in the upper layer and have higher density. Coarse particles are focused on the lower layer, and it is easier to separate the wide-graded particle flow with a significant gradation difference. It is also more difficult to separate the flow of particles.

By analysing the force of a single particle in the flow field, it can be seen that the single particle in the slurry is mainly affected by the pressure gradient force *F*_*p*_, the virtual mass force *F*_*vm*_, the particle acceleration resistance Basset force *F*_*B*_, the particle spin Magnus lift force *F*_*M*_, the Saffman lift force *F*_*s*_ under the pressure gradient, and the dynamic friction force under the collision probability *F*_*L*_.


$$F=F_{s}+F_{B}+F_{M}+F_{p}+F_{vm}+F_{L}$$ (1)

Based on previous research and experimental theoretical analysis, the above mechanical indicators can be analysed and evaluated as follows:

1. The pressure gradient *F*_*p*_ refers to the pressure exerted by the fluid on particles in the fluid flow process [12], which is contrary to the pressure gradient force in the liquid. Therefore, the inertial force of particles is combined with the pressure gradient force.
$$\frac{F_{p}}{m_{p}a_{p}}=\frac{\frac{4}{3}\pi a_{p}^{3}\frac{\partial p}{\partial x}}{\frac{4}{3}\pi a_{p}^{3}p_{p}}=\frac{\partial p/\partial x}{p_{p}a_{p}}$$ (2)
  where $\frac{\partial p}{\partial x}=\rho_{f}a_{f}$; *a*_*p*_ is Particle acceleration; *a*_*f*_ is Fluid acceleration; *ρ*_*p*_ is particle density; *ρ*_*f*_ is the density of slurry;
  Therefore, Formula (2) can be simplified as
$$\frac{F_{p}}{m_{p}a_{p}}=\frac{p_{f}a_{f}}{p_{p}a_{p}}$$ (3)
2. As the particle accelerates, the kinetic energy of the particle itself increases, and as the total kinetic energy of the surrounding fluid increases, the phenomenon that the kinetic energy of the particle drives the fluid to move is equivalent to the virtual mass increase of the particle. Based on hydrodynamic analysis. [13], its virtual mass force is:
$$\frac{F_{vm}}{m_{p}a_{p}}=\frac{\frac{1}{2}p_{f}V_{p}\left(\frac{du_{p}}{dt}-\frac{du_{f}}{dt}\right)}{p_{p}V_{p}\frac{du_{p}}{dt}}=\frac{1}{2}\frac{p_{f}}{p_{p}}(1-\frac{a_{f}}{a_{p}})$$ (4)
3. The Basset force *F*_*B*_ of particle acceleration resistance refers to particle movement in viscous particle flow, and its initial motion force is subject to instantaneous resistance, namely, the Basset force [14]. When the fluid is less turbulent, the Basset force is negligible.
$$F_{B}=\frac{3}{2}d_{p}^{2}\sqrt{\pi p_{f}u}\int_{-\infty }^{t}\frac{\frac{du_{f}}{d\tau }-\frac{du_{f}}{d\tau }}{\sqrt{t-}\tau }d\tau$$ (5)
4. The Magnus lift *F*_*M*_ causes particle collision and friction to the particle rotation force in turbulent flow, and the different properties of soil particles themselves cause particle spin rise [15]. Therefore, according to the analysis of relevant studies, the Magnus lift *F*_*M*_ is:
|FM|=pfApCL2uf−up2 (6)
  where *u*_*f*_ and *u*_*p*_ are the movement velocities of the slurry and particle, respectively. $A_{p}=\frac{\pi d_{p}^{2}}{4}$ is the section area of the particle, and *C*_*L*_ is the rise coefficient.
$$C_{L}=\left\{\begin{array}{c} \Gamma \;(R_{ep}\;\ll \;1)\\ \left(0.4\;\mp \;0.1)\Gamma (550\;<\;R_{ep}\;<\;1600\right)\\ \left(0.16\;\mp \;0.04)\Gamma (1500\;<\;R_{ep}\;<\;3000\right) \end{array}\right.$$ (7)
  where $\Gamma =\frac{d_{p}\omega }{2u_{f}}$ is the dimensionless rotational speed of particles, *ω* is the rotational velocity of the particle, *u*_*f*_ is the relative velocity of particles, and *d*_*p*_ is the particle diameter.
  Bring Formula (7) into Formula (6), we can obtain:
$$\left|F_{M}|=\frac{\rho_{f}\frac{\pi d_{p}^{2}}{4}k\frac{d_{p}\omega }{2\left(u_{f}-u_{p}\right)}}{2}|u_{f}-u_{p}\right|$$ (8)
  The particle rise coefficient of the fluid R_*ep*_ in the range of 1–550 is relatively complex, so it is summarized according to the experimental statistics and rules.
$$F_{M}=\frac{1}{8}\pi d_{p}^{3}\rho_{f}\omega \times (u_{f}-u_{p})$$ (9)
5. Under the pressure gradient, the Saffman lift *F*_*s*_ refers to the pressure at the position with a high velocity that is lower than the pressure at the low-velocity section in the process of fluid flow under the action of turbulence [16]. Therefore, there will be downward and upward flow pressure, which causes particles to rise.
$$F_{s}=1.61{\left(\mu \rho_{f}\right)}^{1/2}d^{2}(u_{f}-u_{p})|{\left|\frac{du_{f}}{dz}\right|}^{1/2}$$ (10)
6. Under the collision probability of dynamic friction *F*_*L*_ is the friction force between soil particles, slurry particles can produce collisions between particles in the horizon of the exchange. However, soil particle collision occurs for random collisions but is present in the fluid flow direction; therefore, in combination with the friction and particle collision probability concept [17], the analysis of particle collision probability.
$$N_{c}=\frac{\sqrt{2}}{2}\pi d_{p}^{2}n_{p}^{2}\langle v_{r}\rangle$$ (11)
  where ❬*v*_*r*_❭ Rangle is the relative speed of motion between particles and *n*_*p*_ is the concentration of slurry.
  Therefore, according to relevant studies, the dynamic friction force of particles sorted in the slurry is
$$F_{L}=C_{L}N_{c}m\bar{v_{p}}=\frac{\left(k_{p}\rho_{b}\right)}{\left(\varepsilon \rho_{s}\right)}\frac{\sqrt{2}}{2}\pi d_{p}^{2}n_{p}^{2}\langle v_{r}\rangle mv_{p}$$ (12)
$$C_{L}=\frac{k_{p}\rho_{b}}{\varepsilon_{s}}$$ (13)
  where *C*_*L*_ is the dynamic friction coefficient; *k*_*p*_ is the dimensionless correction coefficient; and *ρ*_*b*_ is the soil particle density.

Therefore, according to the dynamic balance theory of particles, the centrifugal force of particles in the turbulent flow and the movement orbit of particles in the flow film are analysed. Therefore, the centrifugal force of particles in the slurry flow process is gradually reduced, and gravity plays the dominant force role [18]. Thus, the calculation formula for the ascending law of single particles is as follows.


$$\frac{\pi }{6}\rho_{p}d_{p}^{3}\frac{du_{p}}{dt}=\frac{\pi }{6}\rho_{p}d_{p}^{3}G-\rho_{f}u_{p}G-3\pi \mu d(u_{f}-u_{p})-\frac{\rho_{f}}{\rho_{p}}\frac{a_{f}}{a_{p}}\frac{\pi }{6}\rho_{p}d_{p}^{3}\frac{du_{p}}{dt}-\frac{1}{2}\frac{\rho_{f}}{\rho_{p}}\left(1-\frac{a_{f}}{a_{p}}\right)\frac{\pi }{6}\rho_{p}d_{p}^{3}\frac{du_{p}}{dt}+\frac{1}{8}\pi d_{p}^{3}\rho_{f}\omega (u_{f}-u_{p})$$ (14)

Simplification, the differential equation of the upward migration motion of a single particle is as follows:

$$u=\frac{2(\rho_{p}-\rho_{f})}{2\rho_{p}+\rho_{f}}G-\frac{36\mu (u_{f}-u_{p})}{(2\rho_{p}+\rho_{f})d_{p}^{2}}-\frac{\rho_{f}a_{f}}{2\rho_{p}+\rho_{f}}+\frac{3}{2}\frac{\rho_{f}}{2\rho_{p}+\rho_{f}}\omega (u_{f}+u_{p})$$ (15)

By analysing the differential equation of particle motion in the process of particles settling in the above compound force field, the dynamic equation that the rotating centrifugal field can strengthen the radial separation process of particles is mainly based on the density difference. In the direction of particle suspension, the centrifugal force and fluidization water resistance act as a reaction, but they are not reactive forces in essence. To find the best adaptation of centrifugal force and medium resistance and achieve accurate stratification, the flow of separation medium (the velocity and acceleration of disturbance) plays a crucial role in the movement and stratification effect of the particle group.

### 2.2 The separation mechanism of slurry particle groups under disturbance

#### 2.2.1 Markov chain model

Although the movement position and direction of a single particle in the slurry are directional due to the constraints of the flow field, the particles have randomness in the movement of the particle swarm. In the gradation process, due to the collision of particles and other reasons, the mismatched particles will be mixed in the stratification of particles, related to the hydrodynamics of the fluid, particle gradation, shape, etc. After experimental analysis, it is found that the change in disturbance force and flow state water pressure will affect the probability of particle mismatch. The disturbed manure is thus divided into three layers, namely, the sediment layer, the separation layer and the starting layer. In addition, the particle exchange of the three layers is a random event. The Markov chain model was used to dynamically predict the migration rule and sorting state of particles in the flow field. In this paper, the particles were set into N Markov chains according to their size and density to describe the migration probability of the particle group at the moment of flow [12].


$$P(X_{n+1}=j\left|X_{n}=i\right.)=p_{ij}(n)(i,j=1,2,\ldots ,N)$$ (16)

However, in the flow field, the probabilities of particulate movement and retention in different layers are different. Therefore, in terms of the direction of particle migration, the four regions in the slurry all migrate upward. However, when the particles pass through these three regions, there may be enrichment at different locations in different gradations. The most important effects of enrichment are the characteristics of the particles themselves and the magnitude of the dynamic force. Therefore, when the particles move from the bottom starting layer to the separation layer, they need to pass the starting layer and the sorting layer [18], and two events need to occur [19–21], i.e.

$${\text{P}}_{13}={\text{P}}_{12}*{\text{P}}_{23}$$ (17)

Where P_12_ = P{u_p_<u_r1_}; P_12_ = P{u_p_<u_r2_}

#### 2.2.2 The numerical model for separating the particles in the group

According to the distribution law of each particle swarm, it is related to the initial rising speed and initial density of particles, so the initial density of particles with different sizes is determined first [13].

$$S=\left|\begin{array}{ccc} S_{1,1} & & S_{n,1}\\ & S_{i,j} & \\ S_{1,n} & & S_{n,n} \end{array}\right|$$ (18)

where i is the particle size, *j* is the particle density, and *S*_*i*,*j*_ is the initial density of particle group i layer j. In the process of sorting, the possibility of different particle groups entering the separation zone is determined by the selectivity function *f*, so the possibility of each particle group entering is calculated as follows:

$$f=\left|\begin{array}{ccc} f_{1,1} & & f_{n,1}\\ & f_{i,j} & \\ f_{1,n} & & f_{n,n} \end{array}\right|$$ (19)

where i is the particle size, j is the particle density, *f*_*i*,*j*_ is the density j and the probability of the particle group entering the separation zone; f is a function that positively correlates centrifugal force, slurry water pressure, particle density, particle strength, etc. When particles are lifted, their final speed should be greater than the final speed limit of each layer.


$$u_{p}=\frac{d^{2}}{18\mu }(\delta_{p}-\delta_{f})G$$ (20)

Critical particle levels from underselection and separation areas are.


$$u_{r1}=\frac{\frac{n_{1}}{n_{1}+n_{2}}\cdot u}{2\pi rl}=k_{1}u$$ (21)

The critical speed of the sorting layer and the starter layer is.

$$u_{r2}=\frac{\frac{n_{2}}{n_{1}+n_{2}}\cdot u}{2\pi rl}=k_{2}u$$ (22)

where *n*_1_ and *n*_2_ are n values of the starting layer and sorting layer, respectively.

$$n=K{\left(\sin\;\alpha \right)}^{0.283}\cdot q^{-0.248}$$ (23)

where K is the Kalman constant, which is approximately 0.4 in the thick water layer of the gravitational field. q is the flow rate per unit width; *α* is the angle of inclination of the interference wheel;

As a result, the likelihood of particles entering the sorting area is.


$$f_{\text{starting}}=P\{u_{p}<u_{r2}\}$$ (24)

According to the results of the research, the velocity of the collision of the velocity of particles in the vertical direction presents a normal distribution, which can be obtained by normalizing the above two expressions.


$$f_{\text{separation}}=P\{u_{r1}<u_{p}\}$$ (25)

According to the search results, the velocity of the collision of the velocity of the particles in the vertical direction has a normal distribution, which can be obtained by normalizing the above two expressions [19].


$$f_{\text{starting}}=P\{u_{p}<u_{r2}\}=\sqrt{\frac{2}{\pi }}\int_{u_{r2}}^{u_{r1}}e^{\left(-\frac{u^{2}}{2}\right)}\text{du}$$ (26)

$$f_{\text{separation}}=P\{u_{r1}<u_{p}\}=\sqrt{\frac{2}{\pi }}\int_{u_{r1}}^{+\infty }e^{\left(-\frac{u^{2}}{2}\right)}\text{du}$$ (27)

After entering each layer, the gradation of each particle group in particle sorting is expressed by the Hadamard matrix [14].


$$\rho_{i,j}=S_{i,j}\times f_{i,j}$$ (28)

$$\rho =\left|\begin{array}{ccc} \rho_{1,1} & & \rho_{n,1}\\ & \rho_{i,j} & \\ \rho_{1,n} & & \rho_{n,n} \end{array}\right|$$ (29)

*ρ*_*i*,*j*_ is the density of particle group I after entering layer j. The calculated *ρ* is the density value after the final enrichment of particles in each layer, and the ratio of particle density in each layer is the particle gradation after the sorting of the layer.

## 3. Sludge flow film separation theory

### 3.1 Separation of the flow film

In the traditional theory of centrifugal separation in a fluidized bed, separation of the flow of thick particles is considered to be a flowing film. In the disturbed fluid, the formed fluid film may be divided into the initial layer, the sorting layer, the pass-through layer and the deposition layer. The flow of the film is considered the suspension and separation of nonuniform particles. Along the section direction of the flow film, the suspended layer and the moving layer of the fluid with a significant concentration gradient not only play the role of sorting but also the deposition of coarse particles. As a result, the separation flow film concept is introduced, and the manure separation model under disturbance conditions is configured. Because the impeller is disturbed to stir slurry particles, the separation flow film is formed within the disturbed range, while in the mechanical and physical disturbance, turbulence produces a flow film because of slurry disturbance. Through the analysis of the traditional fluidized bed model, it can be seen that the disturbance in the slurry leads to an increase in the shear force of the fluid particles, and they are gradually sorted in the suspension. The displacement of turbulent particles is very complex. The movement of the sludge occurs not only at longitudinal velocity but also by shear action, and the shear action gradually decreases as it approaches the sedimentary layer. During the process of disturbance, when the fluid moves relative to the disturbed current, the Coriolis effect will be generated. It induces the inertia of particles in the fluid and transfers the tangential shift. With the fluctuation of this type of energy wave, the particle distribution is uneven, and like the transference and dissipation of wave energy, it shows fluctuation performance. It showed volatility. The thickness of the layer can be calculated from the velocity caused by the disturbing force of the layer and the physical parameters of the mud, as shown in the following equation [7].

$$\frac{u_{h}}{u_{H}}={\left(\frac{h}{H}\right)}^{\frac{1}{n}}$$ (30)

$$u_{h}=\frac{1}{K}\sqrt{gHu_{H}}\left(\ln\;\frac{h}{H}+KN^{\prime }\right)$$ (31)

$$n=K{\left(\sin\;\alpha \right)}^{0.283}\cdot q^{-0.248}$$ (32)

$$q=u_{h}H=\frac{\rho_{f}g\sin\;\alpha }{3\mu }H^{3}$$ (33)

$$H=\sqrt[{3}]{\frac{3g\mu }{\rho_{f}g\sin\;\alpha }}$$ (34)

where H is the total thickness of the flow film; *u*_*h*_ is the velocity at the center point h from the disturbed runner; and *u*_*H*_ is the disturbed speed of the disturbed wheel. The n value increases with increasing Reynolds number and decreasing flow film thickness, and the total thickness of the flow film is the most sensitive. g is the acceleration of gravity; K is the Kalman constant, which is approximately 0.4 in the thick water layer of the gravity field. *N*’ is the Prantle number, which takes the value *N*’ = 11.6 in the disturbed slurry; Q is the flow rate per unit width; *α* is the tilt angle of the disturbance wheel; *μ* is the viscosity of the slurry; and *ρ*_*f*_ is the density of plasma before disturbance.

### 3.2 Distribution characteristics of slurry flow film

**Fig 2:**
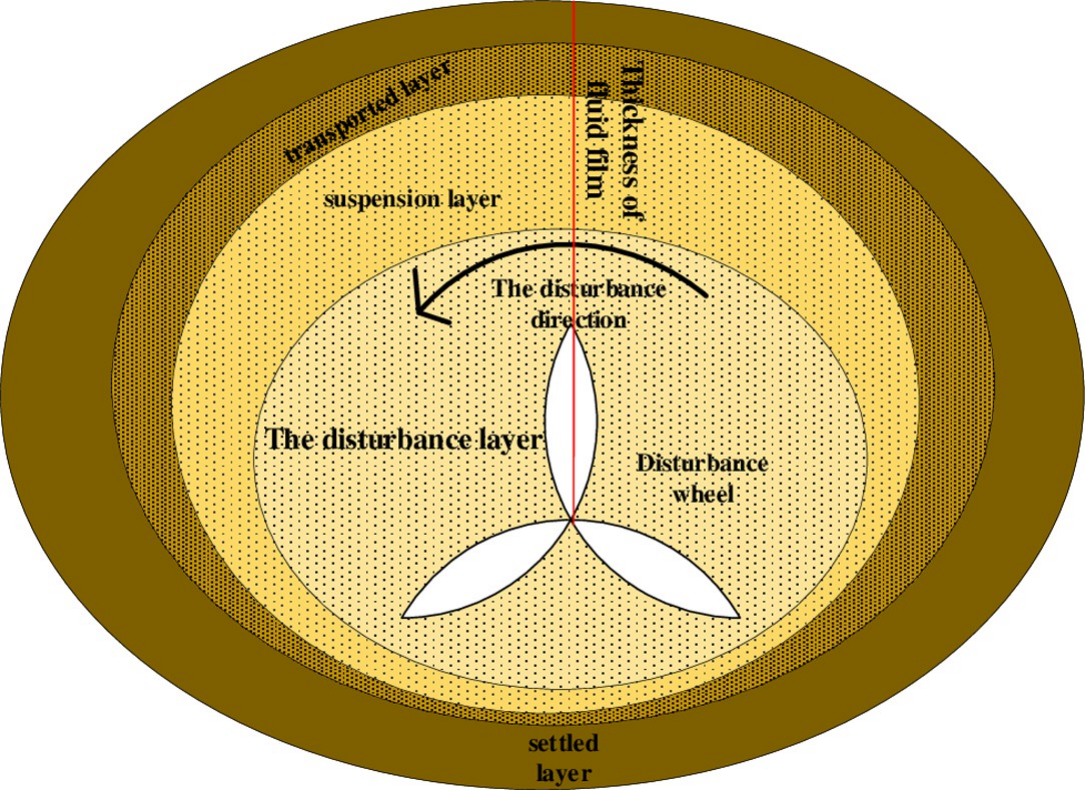
Model diagram of slurry flow film structure under physical disturbance.

The structure distribution characteristics of the flow film can be calculated by the Kalman-Pudelain velocity distribution formula (Eq 1) [1]. Based on the movement of the flow film slurry caused by turbulence, the analysis of the particle concentration in the convection film shows that the concentration in the upper part of the flow film is small, while the concentration in the lower part is large, the overall distribution is elliptical, and the concentration varies greatly. Analysis of the film forming law of the circulating film formed in the process of disturbance of fluid agitation (as shown in Fig 2). The movement of the flowing membrane changes, and the lower part of the membrane is larger than the upper part of the membrane, so the deposited layer in the lower part is larger. After passing through the disturbed layer, the particles form the suspended layer of particle sorting, a passing layer for inertial force and a deposited layer for gravity under the action of turbulence. The suspended layer of separated particles has a great influence on the upper part of the disturbance layer [20]. According to the gravity and the shear force of particle flow in turbulence, the analysis shows that the effective weight of the particles and the shear force on the cross section after deep disturbance can be calculated by the mechanical balance, and the membrane model can be divided into Fig 1 by combining the results from the associated formulas.


$$\frac{\rho_{p}}{\rho_{f}}={\left(\frac{\frac{H}{h}-1}{\frac{H}{a}-1}\right)}^{\frac{v}{K\sqrt{gHi}}}$$ (35)

*ρ*_*p*_ is the particle flow density of the separation membrane; *ρ*_*f*_ is the density of the slurry; kg/m^3^; H is the total thickness of the flow film; H is the distance from the disturbance center, *v* is the particle sedimentation rate; K is the Kalman constant; and $\sqrt{ghi}$ is the friction velocity.

The concentration of graded particles is *ρ*_*f*_ According to the comparison between Eqs (29) and (15), it can be seen that with the disturbance region advancing outward [21], the particle concentration serves as the flow film distribution of the slurry. Based on the literature review [22–24], the following can be found:

$$\rho_{p}=\left\{\begin{array}{l} \rho_{\text{i}}\;Settled\;\text{layer}\;\text{density}\\ 0.8\rho_{f}\;\text{Suspended}\;\text{layer}\;\text{density}\\ \rho_{f}\;\text{Disturbance}\;\text{layer}\;\text{density} \end{array}\right.$$ (36)

*ρ*_*i*_ is the fine particle deposition density. Depending on the density analysis, the limits of the various flux films and the location of the disturbance centre are as follows:

$$\text{h}=\left\{\begin{array}{l} \frac{H}{\left(\frac{H}{\alpha }-1\right){\left(\frac{\rho_{p}}{\rho_{i}}\right)}^{\frac{v}{K\sqrt{gHi}}}}\;Settled\;\text{density}\\ \frac{H}{\left(\frac{H}{\alpha }-1\right){\left(0.8\right)}^{\frac{v}{K\sqrt{gHi}}}}\;\text{Suspended}\;\text{layer}\;\text{density}\\ \frac{H}{\left(\frac{H}{\alpha }-1\right)}\;\text{Disturbance}\;\text{layer}\;\text{density} \end{array}\right.$$ (37)

## 4 Computation and discussion

Based on the similar experimental data in the existing literature, the rationality and accuracy of the formula are tested in this paper. Then, the discrete element simulation software PFC is used to simulate the particle flow of graded particles under different disturbance forces and different graded particles under the same disturbance forces, and the film-forming law of micro-state of particles under complex physical disturbance conditions is analyzed, so as to further verify the deviation of the formula.

### 4.1 Parameters and boundary conditions of numerical model of physical disturbance

**Fig 3:**
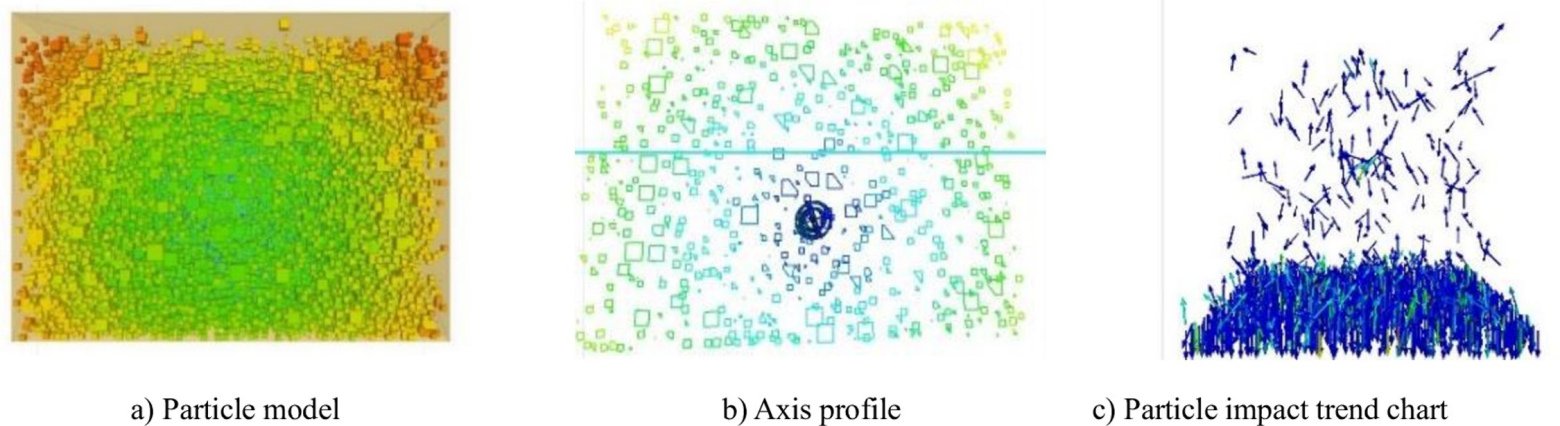
Numerical simulation model. a) Particle model, b) Axis profile, c) Particle impact trend chart.

In this paper, a square box with an experimental groove of 600 mm × 600 mm × 600 mm is constructed by using the constraint wall. As shown in Fig 3A, a fan-shaped disturbance wheel with a diameter of 100 mm is automatically generated by using the model import function of PFC and the CAD model, and the fan-shaped disturbance wheel is generated by the constraint wall with a normal stiffness of 4× 10^10^ N/m. Because the gradation ratio of particles is limited by gradation, particles with different sizes randomly exist in the square box. And after the stable flow field structure is formed under the disturbance condition, PFC software collects the gradation proportion of particles with different sizes in different layers, and records the central axis cross-sectional cloud diagram as shown in Fig 3B (recording the distribution of particles in two-dimensional state and the migration curve of generated particles 1) and the trend diagram of particle collision pressure caused by disturbance as shown in Fig 3C (the cloud diagram mainly shows the trend and magnitude of collision force between particles by arrows of different colors). In order to analyze the sorting law of simulated particles, the basic physical properties of particles used in this paper are all rigid materials. The parameters are shown in Tables 1–3.

**Table 1:** The first simulated particle gradation.

Particle size (mm)	0.074–0.1	0.1–0.12	0.12–0.15	0.15–0.3
Numbers	2500	2500	2500	2500

**Table 2:** Standard configuration of the model calculation parameters.

parameter	u	C	f	*ρ*_0.074–0.1_ (g/cm^3^)	*ρ*_0.1–0.12.1_ (g/cm^3^)	*ρ*_0.12–0.15_ (g/cm^3^)	*ρ*_0.15–0.30_ (g/cm^3^)	*ρ*_*f*_
	(Pa·S)	(KPa)	(KPa)					(g/cm^3^)
Number	0.03	1.4	0.3	1.52	1.72	1.85	1.95	1.82

The disturbance speed was set as 10 r/min,30r/min, 50 r/min, and 70 r/min.

**Table 3:** Parameters of different disturbance forces.

The disturbance rotational speed	10r/min	30 r/min	50r/min	70 r/min
*u*_*f*_ (mm/min)	0.64	2.14	4.86	9.17

### 4.2 Study of the separation law of films at different levels of disturbance

In this section, the grading results of the indoor model test of sandy silt finished by Jia Jingwen [19] are proposed. In this experiment, the Yellow River Delta silt (the initial gradation of the soil sample is 5 b) is used for disturbance experiment. In the experiment, the disturbance force is 0.5 kPa, the disturbance velocity $\bar{V_{b}}$ = 0.06 m/s in the medium and low disturbance state, 0.2 kPa in the high disturbance state and 0.08 m/s in the disturbance velocity ($\bar{V_{b}}$). By comparing the formula with the experimental data, the gradation analysis result of the formula is further verified.

#### 4.2.1 Computation results

**Fig 4:**
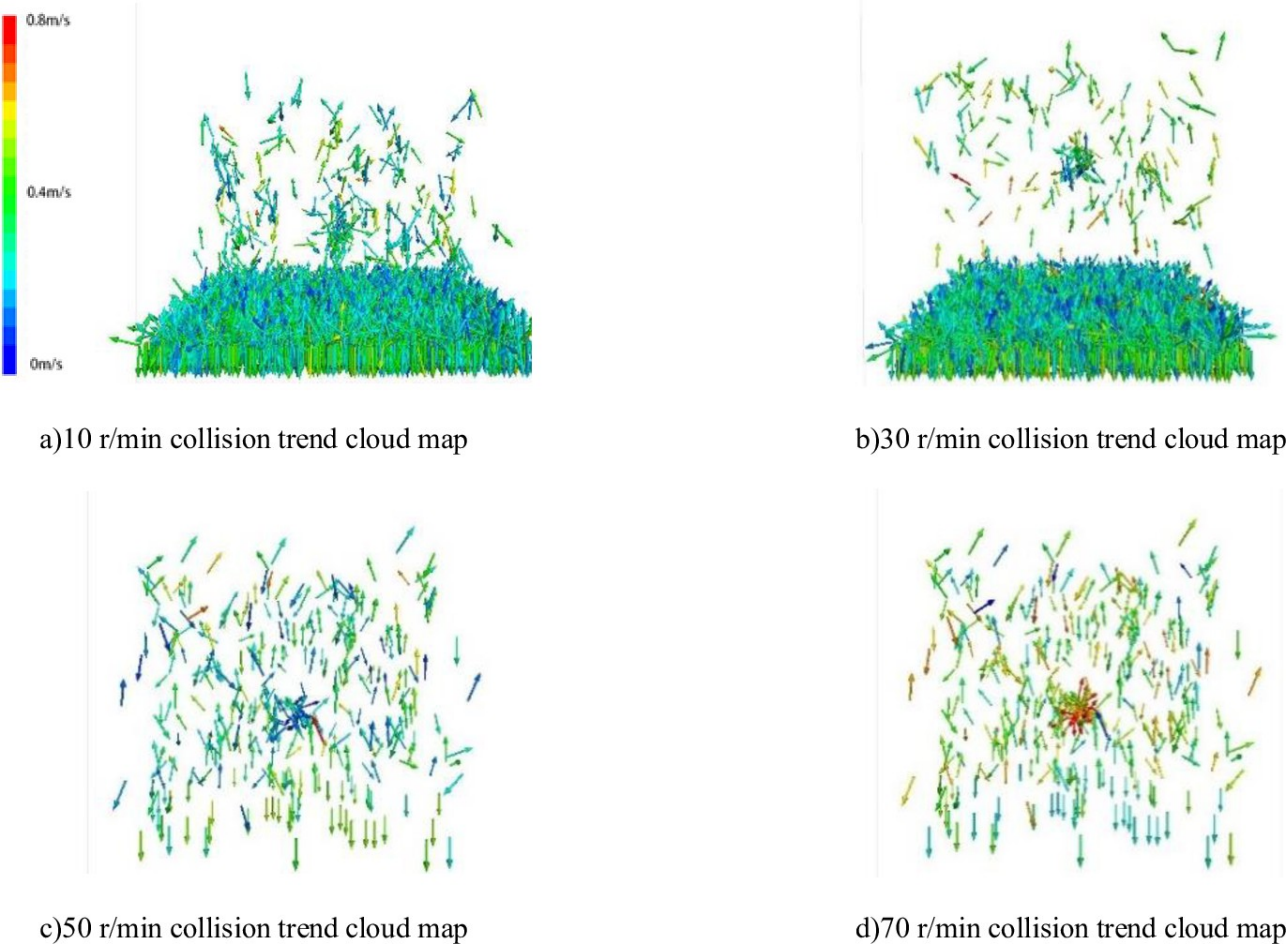
Collision trend cloud map under different disturbance conditions. a)10 r/min collision trend cloud map, b)30 r/min collision trend cloud map, c)50 r/min collision trend cloud map, d)70 r/min collision trend cloud map.

**Fig 5:**
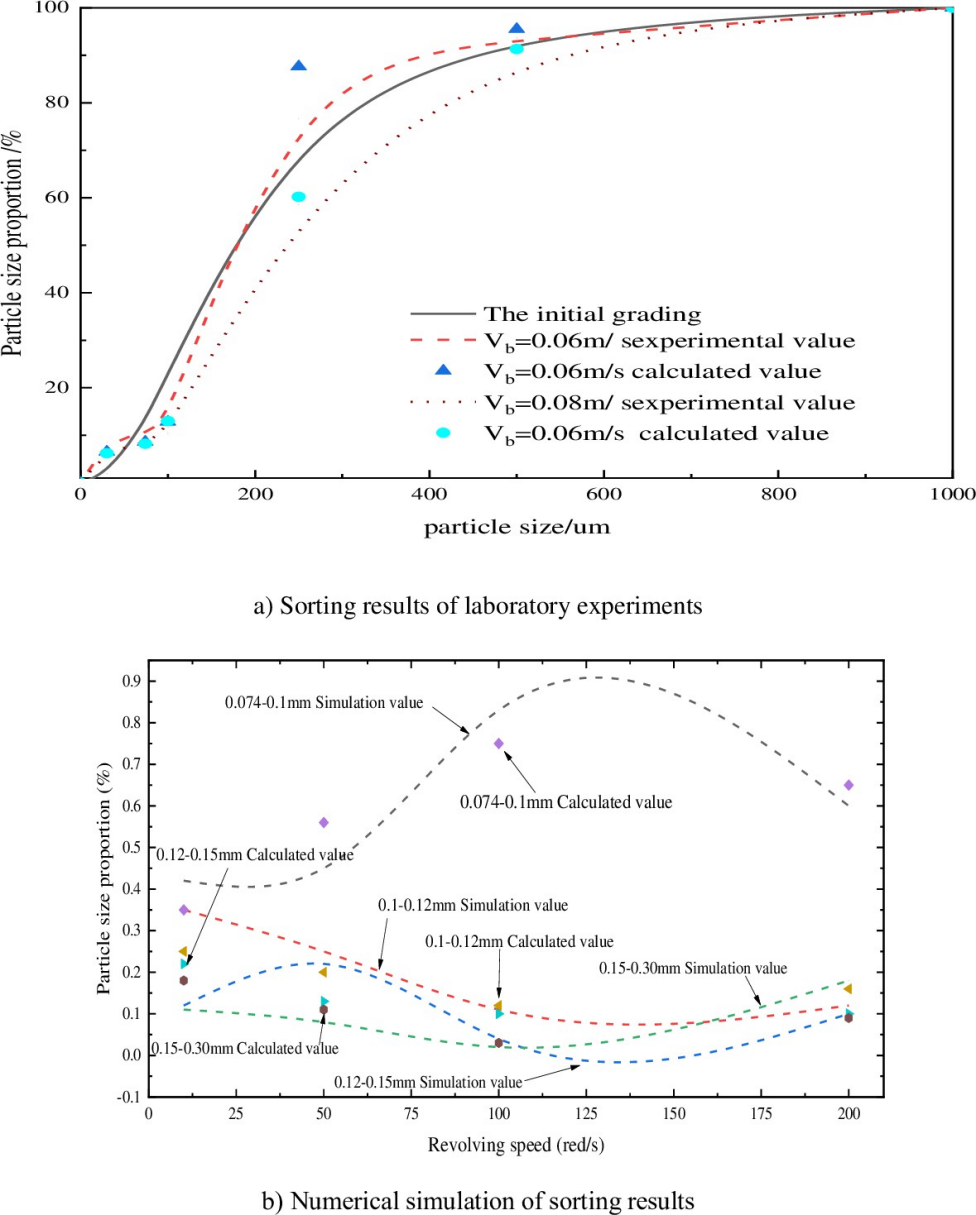
Comparison of separation rules of surface sediments under different disturbance velocities. a) Sorting results of laboratory experiments, b) Numerical simulation of sorting results.

The numerical simulation software experiment will set different impeller disturbance rates as shown in Table 3 and analyze the flow film formation rule of particle flow under different disturbance forces and the particle exchange rule between different layers in the flow film. Since the probability of particle collision increases with the increase of disturbance force, this paper analyzes the cloud picture of particle collision trend during particle disturbance by particle flow simulation and analyzes the formation mechanism of particle flow film in mud under different disturbance forces, as shown in Fig 4. According to formula (30) and the calculation results of numerical simulation, the distribution of particles size distribution in the later stage of disturbance is shown in Fig 5B).

In the comparative analysis of laboratory test data, it can be seen that the increase of disturbance speed can effectively improve the gradation state of soil compared with the initial gradation, when the disturbance speed $\bar{V_{b}}$ = 0.08 m/s and $\bar{V_{b}}$ = 0.06 m/s, Compared with the initial d _50_ value of 180 um, the d _30_ value of soil has increased to 350 um, and compared with the d _50_ value of $\bar{V_{b}}$ = 0.06 m/s, the particle gradation curve is mild, and the dispersion degree of the calculated value is not high, and the deviation is large only when the particle size exceeds 200 um, which is due to the complex field influence on the particle dispersion process of indoor experiments. Due to the gravity and disturbing force in the formula, the migration of coarse particles is not obvious in the actual experiment, but the difference between the formula-level ratio and the experimental value is only about 5%, and the accuracy can meet the calculation requirements.

#### 4.2.2 Discussion

According to the action of different disturbing forces, the hydrodynamics of the particles produced are different, the analysis of simulation and calculation data, if the disturbance force is small, such as at 10 r/min, the variation trend of particle collision generated by disturbance is small, and the flow film generated by slurry is thin, with the proportion of 0.074–0.1 mm being only 42%. By comparing Fig 4A) with Fig 4B), it can be seen that the separation of fine particles at low rotational speed is not obvious because the pressure of the runner is consistent and the disturbance force fails to produce a wide range of flow film structures for particles between slurries. Calculation results and simulation results for surface particle gradation in Fig 5 are analysed. Particulate separation was low under conditions of 10 r/min and 30 r/min. Particle separation was weak at 10 r/min and 30 r/min. When interference power is larger, however, especially when more than 50 r/min, calculated by the formula (25) is 0.15–0.3 mm of *u*_*p*_ velocity than sorting the maximum velocity of *u*_*r*1_, increases the probability of coarse particles into the upper sedimentary, therefore on the basis of particle collision trends in Fig 4C) compared with Fig 4D), as the disturbing force increases the mass collision between particles due to, in the center of outward form velocity gradient, flow membrane structure formation, particle separation efficiency increases gradually, and according to the analysis of Figs 4 and 5 although disturbing force can produce can make the slurry flow membrane, However, when the rotating speed exceeds the maximum flow velocity formed by the slurry flow film, that is, when the slurry speed *u*_*f*_ makes the coarse particles reach the boundary *u*_*p*_ of the bedding layer, the particles in the flow film will cause a large number of coarse particles to enter the suspension zone, resulting in a decline in the separation efficiency. Therefore, the analysis of agitating force and sorting efficiency shows that there is an extreme value of particle sorting, the optimal perturbation sorting speed exists for different graded particle flows, and the optimal perturbation sorting speed for the first type of particle grading is 50 r/min.

### 4.3 Study on the separation precision of the various size distribution widths

**Fig 6:**
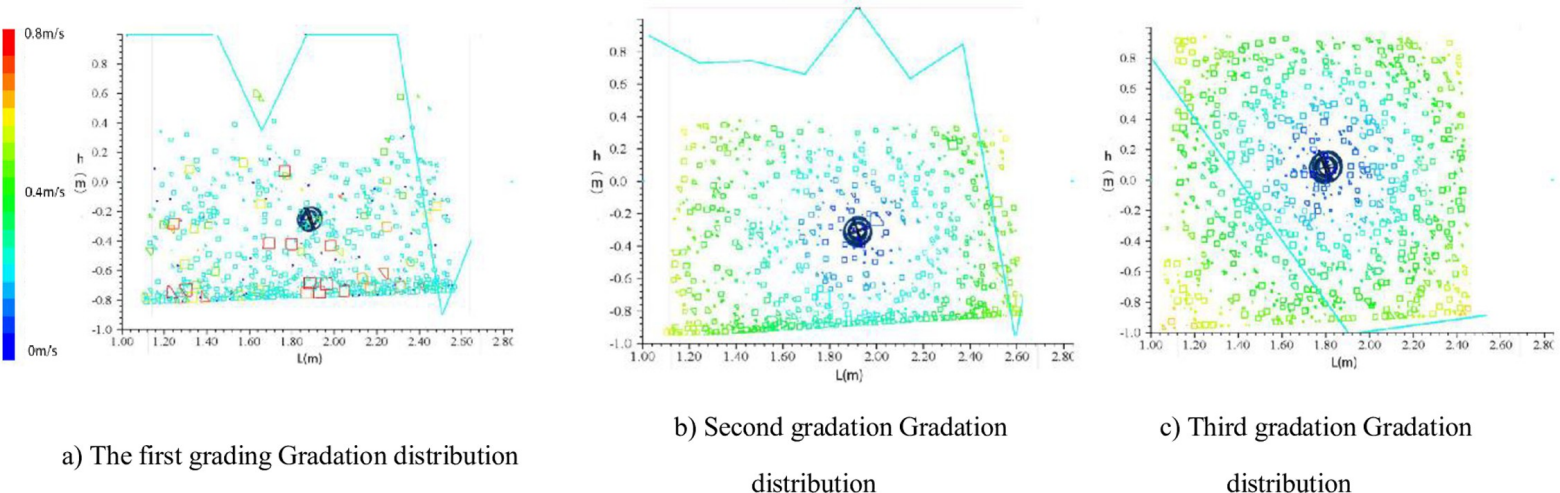
Gradation distribution after disturbance and migration distance record of particle 1. a) The first grading Gradation distribution, b) Second gradation Gradation distribution, c) Third gradation Gradation distribution.

**Fig 7:**
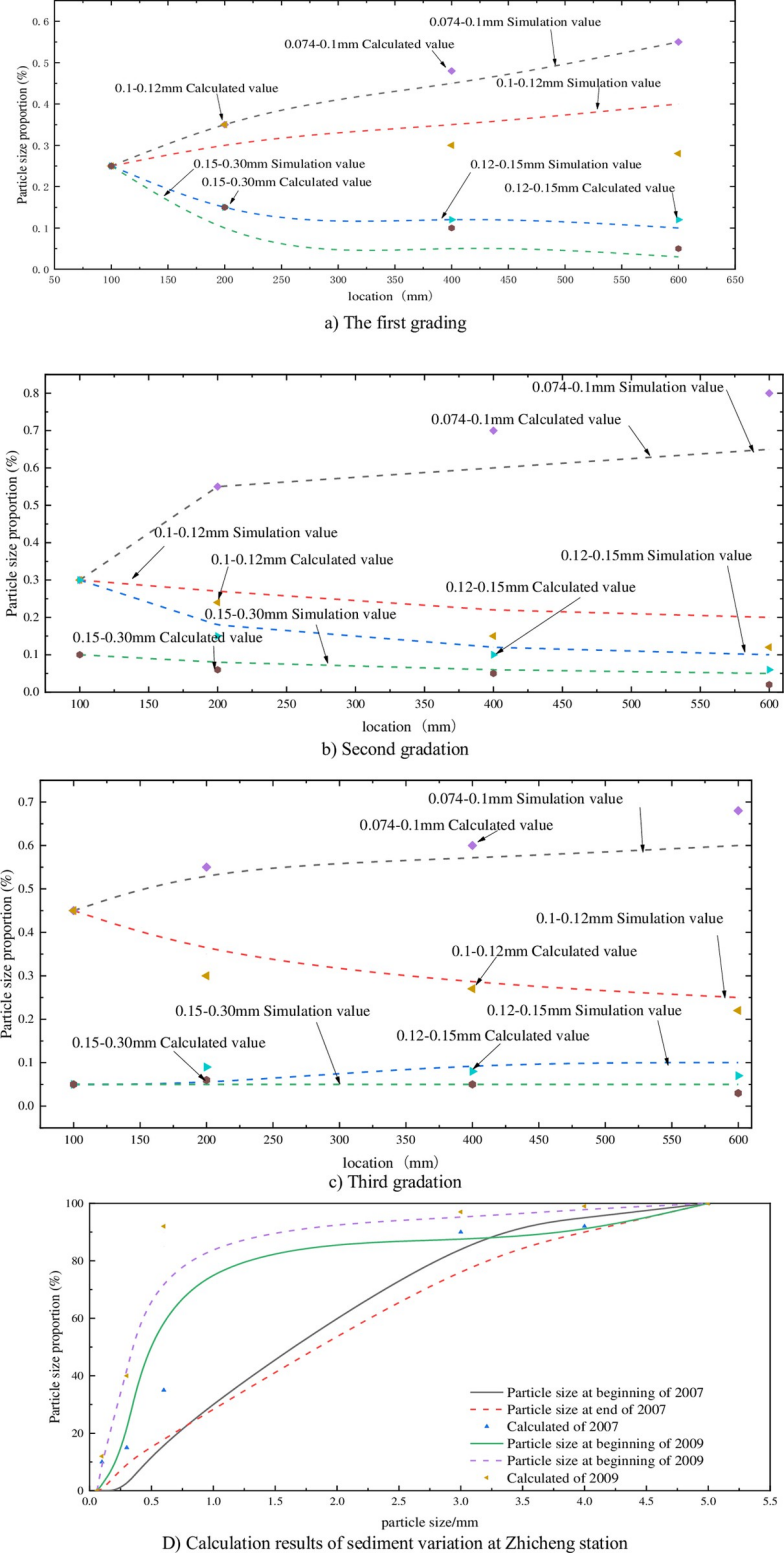
Analysis diagram of analog and computational gradation distribution. a) The first grading, b) Second gradation, c) Third gradation, D) Calculation results of sediment variation at Zhicheng station.

According to theoretical analysis, the surrounding separation power is the same for particles with the same particle size, but if the particle size distribution of the relatively concentrated particle flow is narrow, according to the analysis of formula (29), the final velocity of particles is concentrated. According to the difference in particle gradation, the particle gradation is divided into particle gradation 1 and particle gradation 2(as shown in Tables 4 and 5)., and the best velocity in section 4.2 is 70 r/min. As a result, the numerical simulation method is used to analyse the probability of particle size distribution, as shown in Fig 6. This section selects the hydrological data of Zhicheng Station [19] in the lower reaches of the Three Gorges Project. Due to the construction of the Three Gorges Dam, the river sand in the downstream riverbed cannot be fully supplemented. Therefore, in this paper, the wide gradation condition in 2007 and the narrow gradation condition in 2009 are selected, and the changes of the gradation at the beginning of the year and the gradation at the end of the year after one year of scouring are shown in Fig 7D). Due to the construction of the Three Gorges Dam, the annual average velocity of the Yangtze River is relatively stable at around 3. The gradation data at the beginning of the year and the gradation data at the end of the year are calculated by this formula, as shown in Fig 7D), and the sorting accuracy of this formula for different grain gradations is further analyzed.

**Table 4:** The second type of particle grading.

Particle size (mm)	0.074–0.1	0.1–0.12	0.12–0.15	0.15–0.3
Numbers	3000	3000	3000	1000

**Table 5:** The third type of particle grading.

Particle size (mm)	0.074–0.1	0.1–0.12	0.12–0.15	0.15–0.3
Numbers	4500	4500	500	500

#### 4.3.1 Computation results

According to the analysis of the riverbed gradation data at the beginning and end of 2007 (as shown in Fig 7D), when the grain gradation was wide and when the grain gradation was narrow in 2009, it can be seen that when the grain gradation on the riverbed surface was wide in 2007, the content of fine particles is seriously lost, and d _30_ increases from 1.75 mm to 2.0 mm, while in 2009, due to river erosion, the riverbed gradation was narrow and the particles are fine. The d _50_ is only 0.45 mm, but after scouring, the d _30_ of the year-end gradation is reduced to 0.25 mm, indicating that the scouring force is higher than the starting speed of coarse particles. Comparing the calculated value of the formula, the calculated value of the formula accurately analyzes the trend of particle loss caused by rotating speed, and the calculated loss is slightly higher than the experimental value, with the maximum deviation of only 0.23. Therefore, the formula of separation and deposition in this paper can be applied to different disturbed environments and can reflect the separation law of particle size distribution under disturbed conditions.

According to Fig 6, after the particle flow disturbance is stable, the slurry with gradation 1, 2 and 3 concentrations coagulate and is disturbed, and after disturbance, the particle gradation of 100 mm, 200 mm, 400 mm and 600 mm is analysed from bottom to top. The disturbed particles showed different degrees of deposition, and the separation and deposition of coarse particles were evidently concentrated in the background. Analysis of the 3rd-order distribution showed little change in the range of particle distributions. Analysis of the gradation distribution has shown that particle size tends to decrease at the apex and increase at the bottom. When the disturbing force disturbs the stability of the slurry, record the deposition curve of surface particle 1 and the final deposition state of particles by simulation, as shown in Fig 6.

#### 4.3.2 Discussion

In the first step, the disturbance is more sufficient, the content of coarse particles is high, and the depositing effect of particles is evident. From the migration curve of particle 1, the disturbed particles will continue to drift upwards after deposition, while the secondary and tertiary particles are not suspended under stirring because of their dense and fine particles after deposition, which indicates that particle size distribution is an important condition for producing flow film under the same disturbance condition. Based on the analysis of Figs 1 and 7, the calculation of particle grading was compared with the simulated distribution law, and the separation efficiency (the ratio of fine particles before and after separation) was taken as the main index. The most obvious sorting effect is the first gradation, and the simulated value is smaller than the calculated value, which is also because the simulated particle-free collision causes the particle deposition efficiency to be smaller than the result of the analytical solution, but the overall trend is relatively consistent.

## 5 Conclusion

In this paper, through the concept of hydrodynamics, the disturbance separation law of dense particle flow is studied and combined with particles comparing the simulation and experimental data with the calculation results, the following conclusions are drawn.: (1) The relationship between centrifugal force (fluid velocity and acceleration) and medium resistance (particle density and cohesion) is analyzed by using the liquid film theory of fluidized bed. The particle migration probability between different layers of liquid film is calculated by using Markov chain probability model, and a model of dense particle flow disturbance separation liquid film is established, which can be accurate. (2) The flow film presents a suspended layer for particle separation, a moving layer dominated by inertia force and a deposition layer dominated by gravity under the action of turbulence, and with the increase of disturbance force, the thickness of the suspended layer of the flow film increases, and the upper deposition layer first increases and then decreases. (3) The fixed particle size distribution corresponds to the optimal disturbing force *u*_*r*2_. When *u*_*p*_ > *u*_*r*2_, the structure of the flow membrane is destroyed, resulting in the reduction of the separation efficiency. The narrower the particle size distribution is, the greater the influence of the precision of disturbing force on the separation. This provides a basis for the separation of dredger fill with physical disturbance and the study of sedimentation law. This study provides a theoretical basis for studying the separation, physical disturbance and deposition law of dredger fill, as well as the process of improving the initial strength of soil layer in dredger fill engineering in coastal areas.

## Supporting information

S1 File (RAR)
